# Dementia Incidence in Quebec Over 20 Years

**DOI:** 10.1001/jamanetworkopen.2024.47346

**Published:** 2024-12-02

**Authors:** Claire Godard-Sebillotte, Sanjna Navani, Louis Rochette, Victoria Massamba, Éric Pelletier, Edeltraut Kröger, Isabelle Vedel

**Affiliations:** 1 The Research Institute of the McGill University Health Centre, Montreal, Quebec, Canada; 2 Division of Geriatrics, Department of Medicine, McGill University, Montreal, Quebec, Canada; 3 Institut National de Santé Publique du Québec, Quebec, Canada; 4 Center of Excellence on Aging in Quebec, CIUSS de la Capitale-Nationale, Quebec City, Quebec, Canada; 5 Faculty of Pharmacy, University of Laval, Laval, Quebec, Canada; 6 Lady Davis Institute, Jewish General Hospital, Montreal, Quebec, Canada; 7 Department of Family Medicine, McGill University, Montreal, Quebec, Canada

## Abstract

This cross-sectional study examines the incidence of dementia from 2003 to 2023 in Quebec, Canada.

## Introduction

Projections based on stable dementia incidence indicate that compared with 2019, the number of people living with dementia in 2050 will triple globally.^1^ However, some high-income countries have reported a decreasing dementia incidence.^2,3^ Using a decreasing incidence scenario results in 36% lower projections of people with dementia in 2050 compared with the tripled numbers in the stable-incidence scenario.^1,4^ Describing population-wide incidence trends is imperative for understanding global incidence trends, understanding their impact on future prevalence, and informing public health policy and planning.^1^ We report the incidence of diagnosed dementia in the Quebec population aged 40 years and older between April 1 and March 31 from 2003-2004 to 2022-2023.

## Methods

In a cross-sectional design, we extracted data from health administrative databases, which record most services provided via the public Quebec universal health insurance system. Per a validated algorithm, a person was considered to have received a dementia diagnosis on the day they fully met any of 3 criteria: (1) principal or secondary diagnosis of dementia recorded in the hospitalization file, (2) 3 dementia diagnoses 30 days or more apart within a 2-year period recorded in the physician claims file, or (3) dispensation of a dementia-specific medication (donepezil, rivastigmine, galantamine, or memantine) recorded in the pharmaceutical services file.^5^ Estimates were age-adjusted across 7 bands (40-64, 65-69, 70-74, 75-79, 80-84, 85-89, and ≥90 years), using direct standardization based on the age structure of the Quebec population in 2011.^6^ We evaluated trends in incidence based on graphical assessment. Changes to the physicians claim system in 2016 resulted in fewer diagnostic codes being entered into this file: estimates from 2016 onward likely underestimate the incidence compared with previous years. This study was exempt from ethics review as it was conducted as part of continuous chronic disease surveillance under the oversight of the provincial Ethics Committee of Public Health. This descriptive, cross-sectional study was conducted in accordance with the STROBE reporting guideline. Data analysis was performed using SAS Software, version 7.15 (SAS Institute LLC).

## Results

The Figure shows the crude and age-adjusted incidence of diagnosed dementia in Quebec from 2003-2004 to 2022-2023. The age-adjusted incidence was 4.9 at the start of the study period and 5.0 at the end: it remained relatively stable over 20 years, other than a dip during the peak of the COVID-19 pandemic; despite this considerable stability, the number of people with a new diagnosis of dementia increased sharply, with a 78% relative increase over the study period (from 14 830 in 2003-2004 to 26 335 in 2022-2023).

**Figure.**
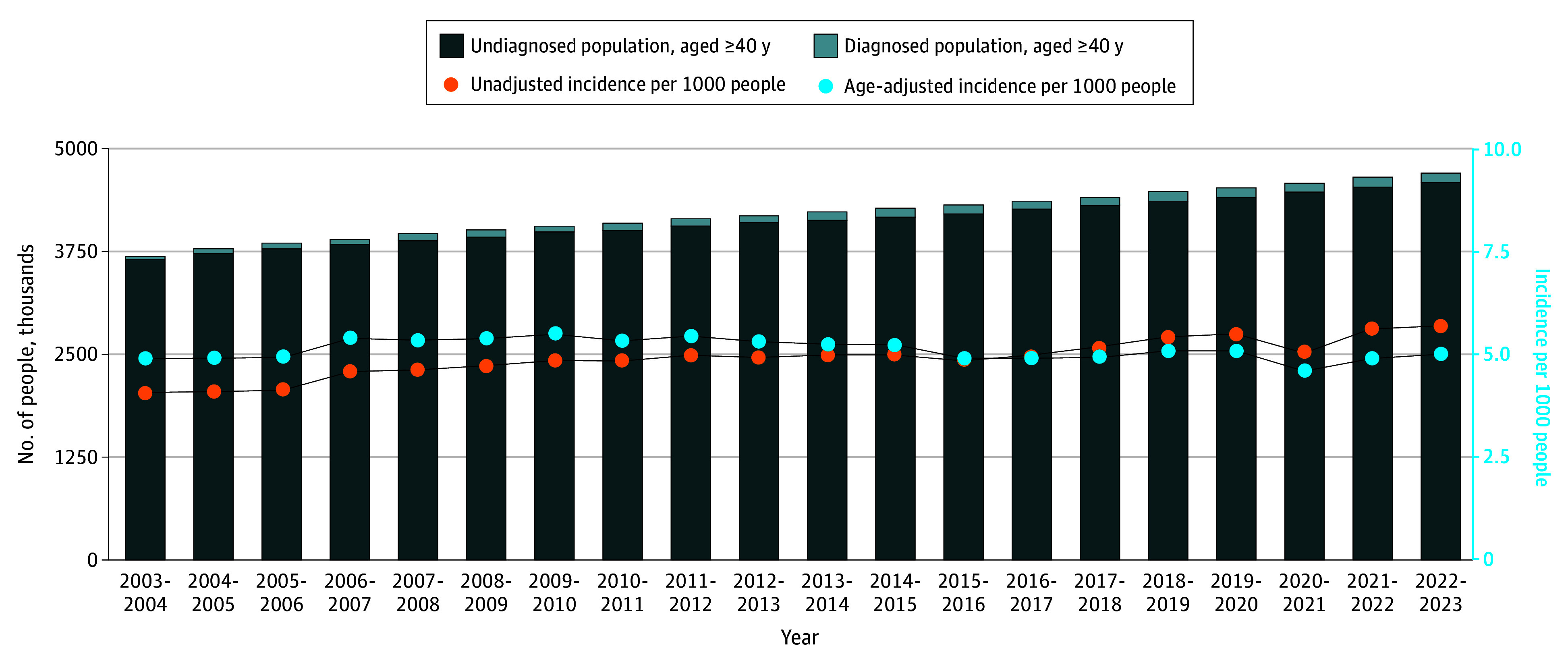
Crude and Age-Adjusted Dementia Diagnostic Incidence in the Population Aged 40 Years and Older in Quebec

## Discussion

The age-adjusted incidence of diagnosed dementia in Quebec was relatively stable from 2003-2004 to 2022-2023. These findings diverge from expectations of a decreasing incidence in high-income countries, including from studies that similarly use electronic medical records, especially since estimates onward of 2016 underestimate the incidence in our data.^1,2,3^

Reasons attributed to decreased incidence include improvements in education, reductions in smoking, and better prevention and treatment of cardiovascular and chronic conditions.^2,3^ It is plausible that the dementia incidence did not decrease in Quebec due to suboptimal public health strategies.

However, considering substantial underdiagnosis in dementia, it is possible that the incidence decreased but that improvements in diagnosis and care reduced underdiagnosis rates and artificially inflated the diagnostic incidence to stable levels. There is a discrepancy between the real incidence of dementia (actual number of cases of dementia) and the diagnosed incidence (number of cases of dementia identified and coded as such in health administrative data). Despite limitations related to dementia diagnosis and identification through the algorithm, changes in the claims system, and evolving diagnostic and prescription standards, our results suggest a lack of relief on the burden dementia places on health systems and their resources.

Given a relatively stable diagnostic incidence of dementia and its implications for future prevalence, Quebec may reach estimated projections of tripling dementia cases as an aging province. Decision-makers and clinicians need to prepare for substantial increases in the number of people with new dementia diagnoses and adapt policies to scale up health care provision and services infrastructure to meet greater demand.

## Notes

Supplement.
Data Sharing Statement
