# Supplementary material for: Dementia Incidence in Quebec Over 20 Years
Source: JAMA Netw Open. 2024 Dec 2;7(12):e2447346. doi: 10.1001/jamanetworkopen.2024.47346 (PMC11612831; doi:10.1001/jamanetworkopen.2024.47346)
Supplement: Supplement. — Data Sharing Statement [file jamanetwopen-e2447346-s001.pdf]

Data Sharing Statement

Godard-Sebillotte. Dementia Incidence in Quebec Over 20 Years. *JAMA Netw Open*. Published December 02, 2024. doi:10.1001/jamanetworkopen.2024.47346

Data
Data available: Yes
Data types: Data (not involving human participants)
How to access data: The data used in the current study are held at the Institut national de santé publique du Québec: data access may be requested from the institution for authorised purposes.
When available: With publication

Supporting Documents
Document types: None

Additional Information
Who can access the data: Researchers whose proposed use of the data has been approved.
Types of analyses: For purposes approved by the INSPQ.
Mechanisms of data availability: After approval of a proposal.
Any additional restrictions: CGS had full access to all the data in the study and takes responsibility for the integrity of the data and the accuracy of the data analysis.
